# Restoration of lysosomal function after damage is accompanied by recycling of lysosomal membrane proteins

**DOI:** 10.1038/s41419-020-2527-8

**Published:** 2020-05-14

**Authors:** Ida Eriksson, Petra Wäster, Karin Öllinger

**Affiliations:** Experimental Pathology, Department of Biomedical and Clinical Sciences, Linköping University, 58185 Linköping, Sweden

**Keywords:** Autophagy, Apoptosis, Lysosomes

## Abstract

Lysosomes are central organelles for cellular degradation and energy homeostasis. In addition, lysosomal membrane permeabilization (LMP) and subsequent release of lysosomal content to the cytosol can initiate programmed cell death. The extent of LMP and available repair mechanisms determine the cell fate after lysosomal damage. In this study, we aimed to investigate the premises for lysosomal membrane repair after LMP and found that lysosomal membrane damage initiated by l-leucyl-l-leucine methyl ester (LLOMe) caused caspase-dependent apoptosis in almost 50% of the cells, while the rest recovered. Immediately after LLOMe addition, lysosomal proteases were detected in the cytosol and the ESCRT-components ALIX and CHMP4B were recruited to the lysosomal membrane. Next, lysophagic clearance of damaged lysosomes was evident and a concentration-dependent translocation of several lysosomal membrane proteins, including LAMP2, to the cytosol was found. LAMP2 was present in small vesicles with the N-terminal protein chain facing the lumen of the vesicle. We conclude that lysophagic clearance of damaged lysosomes results in generation of lysosomal membrane protein complexes, which constitute small membrane enclosed units, possibly for recycling of lysosomal membrane proteins. These lysosomal membrane complexes enable an efficient regeneration of lysosomes to regain cell functionality.

## Introduction

Lysosomes are acidic organelles central for cellular degradation, repair of the plasma membrane, exocytosis, cholesterol homoeostasis and regulated cell death^1–5^. The lysosome contains around 60 different hydrolases, out of which the cathepsins are the most abundant^6,7^. Upon lysosomal membrane permeabilization (LMP), cathepsins are released to the cytosol with subsequent induction of various forms of regulated cell death^5,8–10^.

Maintenance of lysosomal membrane integrity is vital for the cell as LMP can be induced by for example endocytosed pathogens seeking access to the cytosol or by amyloid aggregates, resulting in inflammation, neurodegeneration and cell death^11–14^. Recently, it was discovered that lysosomal damage activates cellular repair mechanisms to protect against LMP-induced cell death. Limited lysosomal damage causes calcium release and a rapid recruitment of the endosomal sorting complex required for transport (ESCRT), that facilitate repair of the lysosomal membrane and thus recovery of damaged lysosomes^15,16^. Unrepairable lysosomal damage activates clearance of damaged organelles by lysophagy. This selective form of autophagy is initiated by galectins that enter the damaged lysosomes and bind exposed lysosomal glycosylated proteins, followed by recruitment of specific E3 ligases to tag the lysosome with ubiquitin^17–19^. An autophagosomal isolation membrane is formed and through fusion with intact lysosomes the damaged organelle is degraded^19^.

Lysosomal membrane stability and thus susceptibility to LMP is dependent on the membrane composition. More than 100 different lysosomal membrane proteins have been identified so far, of which the lysosome-associated membrane proteins (LAMPs) and the lysosome integral membrane proteins (LIMPs) are the most abundant^20^. The mechanism behind LMP is not yet fully understood. However, depletion of SCAV3, the *C. elegans* homologue to human LIMP-II, causes rupture of lysosomal membranes^21^, and knockdown of LAMP1 or LAMP2 sensitises the cell to LMP-inducing drugs^22^. In a previous study, we found that LAMP2 was translocated from lysosomes to the cytosol during LMP-induced apoptosis^23^ raising questions if lysosomal membrane proteins are actively or passively released to the cytosol following LMP. Here, we investigate the premises for lysosomal membrane proteins during lysosomal membrane repair after LMP.

## Results

### LLOMe causes concentration-dependent cell death

**Fig. 1:**
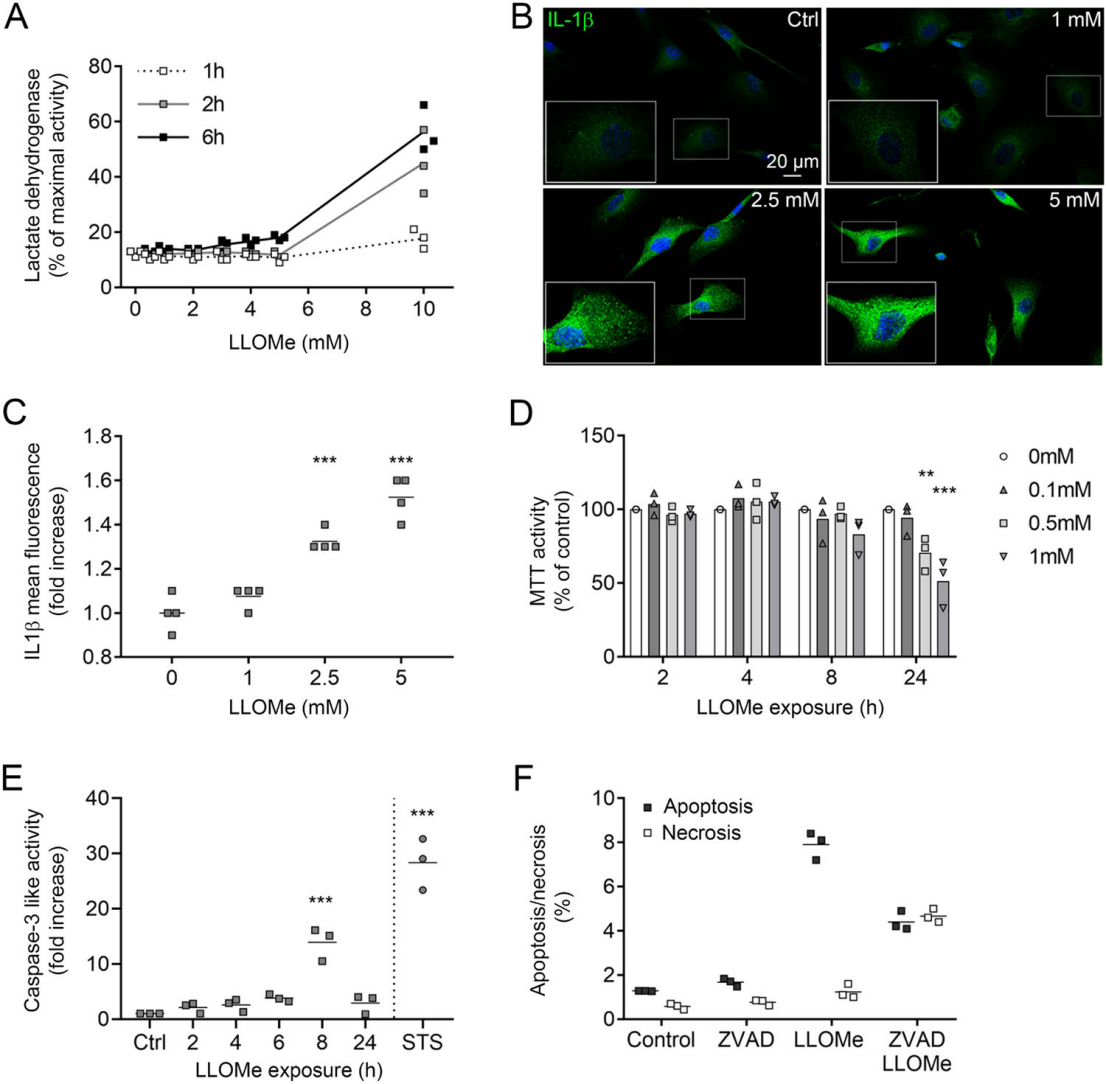
LLOMe induces concentration-dependent apoptosis or necrosis. Human skin fibroblasts were exposed to l-leucyl-l-leucine methyl ester (LLOMe). **a** LDH activity in conditioned medium after exposure to 0.5–10 mM LLOMe for 1–6 h (*n* = 3). **b** Immunostaining of IL-1β (green) and DAPI (blue) in fibroblasts exposed to LLOMe for 2 h. **c** Quantification of IL-1β mean fluorescence in images obtained in **b** (*n* = 4). **d** Cell viability estimated using the MTT assay (*n* = 3). **e** Caspase-3 like activity after exposure to 1 mM LLOMe (0–24 h) or 1 µM staurosporine (6 h, *n* = 3). **f** Percentage of apoptotic and necrotic cells using Annexin V/PI staining in cells pretreated with the pan-caspase inhibitor Z-VAD-FMK (10 µM, 1 h) followed by 1 mM LLOMe for 6 h (*n* = 3). **p < 0.01, and ***p < 0.001 compared to untreated control.

To study lysosomal release and repair mechanisms, we established a cell damaging model using the lysosomotropic agent l-leucyl-l-leucine methyl ester (LLOMe). LLOMe enters the lysosome through receptor mediated endocytosis and is converted by dipeptidyl peptidase I to a hydrophobic polymer with membranolytic activity^24^. Previous studies have interlinked LLOMe-induced LMP and release of cathepsins to the cytosol with activation of the NLRP3 inflammasome, which promotes maturation and release of IL-1β and IL18 and subsequent activation of pyroptosis^25^. In human skin fibroblasts, plasma membrane rupture and release of lactate dehydrogenase (LDH) to the medium was detected at concentrations above 5 mM LLOMe (Fig. 1a). Immunostaining revealed an increased expression of IL-1β after exposure to 2.5 and 5 mM of LLOMe but not at 1 mM (Fig. 1b, c). Thus, to study lysosomal repair mechanisms, LLOMe doses ≤1 mM was used. We detected reduction in viability that was concentration- and time-dependent (Fig. 1d), and preceded by apoptosis, as measured by caspase-3 like activity (Fig. 1e). Staurosporine, a known apoptosis inducer was used as a positive control. By inhibiting caspases using the pan-caspase inhibitor Z-VAD-FMK, the percentage of apoptotic cells was reduced (Fig. 1f).

### LLOMe induces release of lysosomal proteases and translocation of lysosomal membrane proteins to the cytosol

**Fig. 2:**
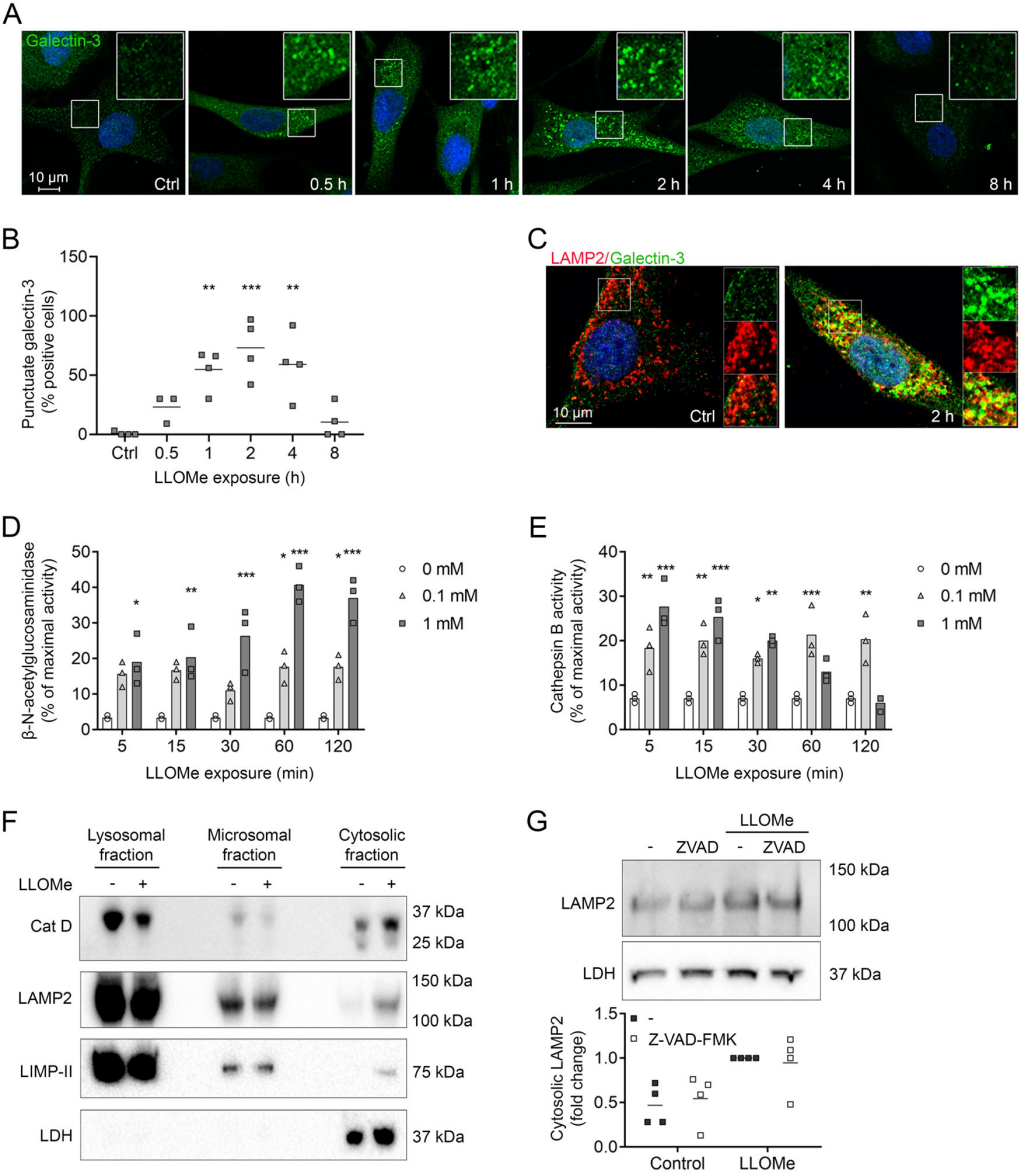
LLOMe-induced apoptosis is preceded by release of lysosomal constituents to the cytosol. Human fibroblasts were exposed to 0–1 mM LLOMe for selected time points. **a** Immunostaining of galectin-3 (green) and DAPI (blue) with **b** corresponding quantitative analysis of cells positive for punctuate galectin-3 staining after exposure to 1 mM LLOMe. Lysosomes in >50 cells from randomly selected areas were quantified (*n* = 4). **c** Immunostaining of galectin-3 (green), LAMP2a (red) and DAPI (blue) in cells exposed to 1 mM LLOMe. **d** Enzymatic activity of N-acetyl-β-d-glucosaminidase in cytosolic extracts (*n* = 3). **e** Cathepsin B activity in cytosolic extracts (*n* = 3). **f** Immunoblot of cell fractions obtained by differential centrifugation from cells exposed to 1 mM LLOMe for 2 h. LDH is used as a cytosolic marker, and cathepsin D as an indicator of LMP. **g** Immunoblot of LAMP2 in digitonin-extracted cytosolic fractions obtained from fibroblasts pretreated with Z-VAD-FMK (10 µM, 1 h) followed by 1 mM LLOMe for 2 h with corresponding quantitative analysis (*n* = 4). *p < 0.05, **p < 0.01 and ***p < 0.001 compared to untreated control.

LLOMe exposure (1 mM) resulted in time-dependent accumulation of galectin-3 in a punctuate pattern that was evident within 30 min and peaked after ~2 h (Fig. 2a, b). The accumulation was mainly lysosomal as galectin-3 puncta colocalized with LAMP2 (Fig. 2c). In addition, a concurrent concentration-dependent release of the lysosomal protease N-acetyl-β-d-glucosaminidase (NAG) to the cytosol was detected. Cytosolic activity of NAG was present already after 5 min of LLOMe exposure, but maximum levels were not reached until after 1 h (Fig. 2d). Noteworthy, the cytosolic activity of cathepsin B increased more rapidly than NAG in cultures exposed to 1 mM LLOMe, and the activity declined within 30 min (Fig. 2e). Considering the molecular weight of the lysosomal proteases, release from the lysosome seems size dependent, since NAG is larger than cathepsin B^26^. To confirm release of lysosomal proteins, immunoblot showed increase of cathepsin D in the cytosolic fraction after 2 h of LLOMe exposure (Fig. 2f). In addition, we confirmed our previous finding^23^ that the lysosomal membrane proteins LAMP2, and also LIMP-II, appear in the cytosolic fraction, following LMP (Fig. 2f). Interestingly, inhibition of caspases had no effect on the levels of cytosolic LAMP2, indicating that the LAMP2 released to the cytosol is not consistent with apoptotic bodies formed due to LMP-induced apoptosis or derived from overall cell decay (Fig. 2g).

### LMP is accompanied by increased level of lysosomal membrane proteins in the cytosol

**Fig. 3:**
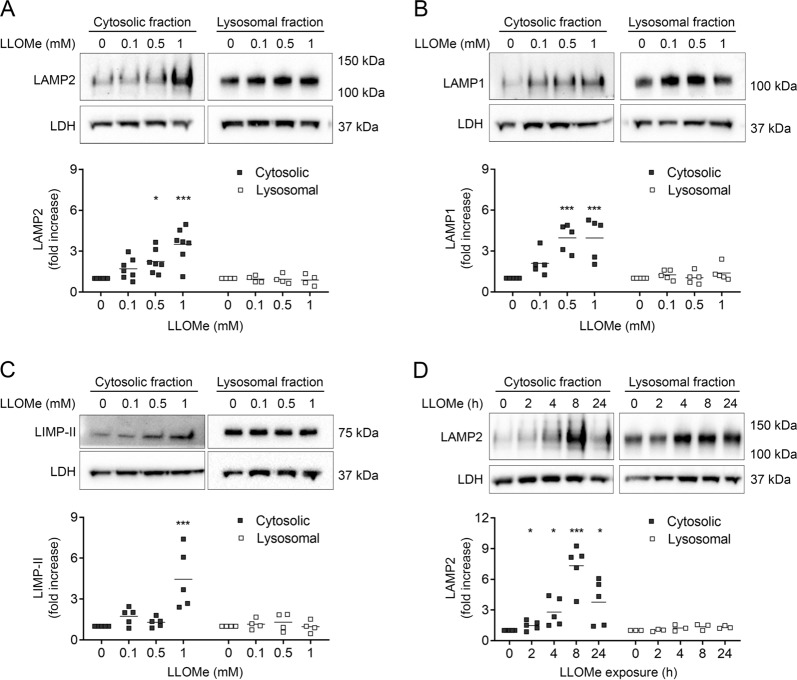
Lysosomal membrane proteins are present in the cytosol after LMP induction. Human fibroblasts were exposed to 0–1 mM LLOMe for 2 h. Cytosolic fractions obtained using digitonin and the remaining pellet (lysosomal fractions) were immunoblotted against **a** LAMP2, **b** LAMP1 and **c** LIMP-II. **d** Time dependence of LAMP2 in cytosolic and the remaining pellet (lysosomal fraction) immunoblotted from cells exposed to 1 mM LLOMe. Representative immunoblots with corresponding densitometric analysis. (*n* ≥ 4), *p < 0.05, **p < 0.01 and ***p < 0.001 compared to untreated control.

To further unravel the relevance of the cytosolic presence of lysosomal membrane proteins, fibroblasts were exposed to increasing concentrations of LLOMe, after which cytosolic and lysosomal fractions were obtained using digitonin extraction. Immunoblotting revealed a dose-dependent increase of LAMP2 (Fig. 3a) and LAMP1 (Fig. 3b) in cytosolic fractions, while no significant alteration was observed in the remaining lysosomal fractions. LAMP1 and LAMP2 both contain a single transmembrane region. We also investigated LIMP-II, a lysosomal membrane protein with two membrane spanning domains and found similar results (Fig. 3c). In our previous study, cytosolic appearance of LAMP2 was found after cisplatin treatment^23^ and to validate the general finding, we investigated if various LMP-inducers and other cell types also presented LAMP2 in the cytosol. Consequently, we found LAMP2 in fibroblasts exposed to the lysosomotropic detergent O-methyl-serine dodecylamine hydrochloride (MSDH) (Supplementary Fig. 1A). Human keratinocytes exposed to UVA or UVB radiation also generated cytosolic LAMP2 (Supplementary Fig. 1B, C). Investigation of the time dependence of cytosolic LAMP2 revealed elevated LAMP2 after ~2 h of LLOMe treatment, and a continuous rise for at least 8 h (Fig. 3d). However, after 24 h the level was reduced in the surviving cells. Staining with Lysotracker showed that LLOMe exposure initially reduced Lysotracker-positive lysosomes, as a sign of lysosomal membrane damage. However, lysosomes recovered as the number of LAMP2 and Lysotracker double-positive lysosomes increased continuously from 4 to 24 h (Supplementary Fig. 1D).

### LAMP2 is released to the cytosol as part of small membrane protein complexes

**Fig. 4:**
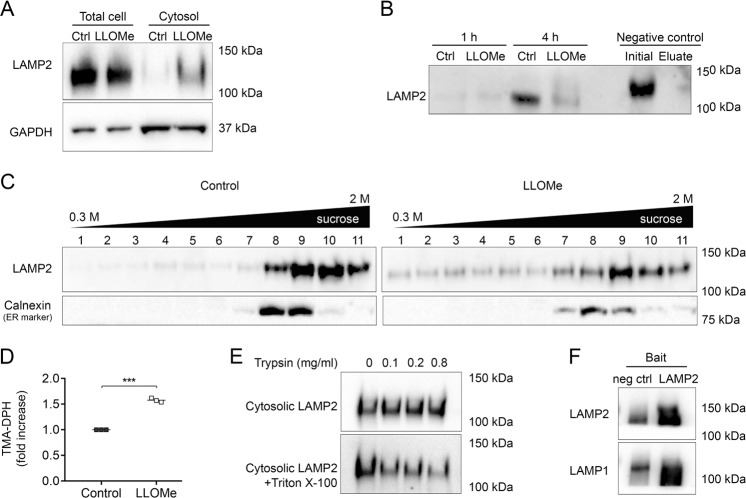
LAMP2 released to the cytosol is part of small membrane vesicles. **a** Immunoblot of LAMP2 in cell lysates and cytosolic extracts using digitonin after exposure to 1 mM LLOMe for 8 h. GAPDH is used as a loading control. Cell lysates were diluted 1:2 compared to cytosolic extracts. **b** Nascent LAMP2 in streptavidin precipitated lysates from fibroblasts exposed to 1 mM LLOMe and co-incubated with a biotinylated methionine analogue for 1 or 4 h. Negative controls incubated without methionine analogue before (initial) and after (eluate) precipitation are included. **c** Sucrose gradient fractions (0.3–2 M) immunoblotted against LAMP2 from cells exposed to LLOMe (1 mM, 2 h). Calnexin is used as an organelle marker. **d** Quantification of membranes in cytosolic extracts using the fluorescent membrane probe TMA-DPH (LLOMe 1 mM, 2 h, *n* = 3, ***p < 0.001). **e** LAMP2 in cytosolic extracts incubated with or without Triton X-100 (1%, 20 min) before subjected to trypsin digestion (100–800 µg/ml, 15 min, LLOMe 1 mM, 2 h). **f** Immunoblot of LAMP1 and LAMP2 in immunoprecipitated cytosolic fractions, using LAMP2 as bait, obtained from fibroblasts exposed to 1 mM LLOMe for 2 h. Isotype IgG is used as a negative control.

As evident in Fig. 4a, only a minor fraction of the total amount of LAMP2 was found in the cytosol after LMP induction. To determine if cytosolic LAMP2 is derived from newly synthesised proteins en route to the lysosomal membrane, cultures were incubated with a methionine analogue for 1 or 4 h, followed by ligation to biotin and precipitation using streptavidin. No increase of nascent LAMP2 was detected upon LLOMe exposure (Fig. 4b). If anything, LLOMe decreased protein synthesis. However, protein synthesis did occur, since a number of biotin-conjugated proteins were detected (Supplementary Fig. 2A), and exposure to the protein synthesis inhibitor cycloheximide for 4 and 24 h completely diminished this (Supplementary Fig. 2B). Moreover, when analysing total amount of LAMP2, LLOMe did not affect the levels, neither with increasing time (Supplementary Fig. 2C) nor concentration (Supplementary Fig. 2D), concluding that the increased levels of lysosomal membrane proteins in the cytosol are not due to protein synthesis to compensate for aberrant lysosomal function. In addition, cytosolic LAMP2 did not seem to shift in molecular weight but, since LAMP2 is heavily glycosylated, estimation of protein size on immunoblot is precarious.

Owing to its hydrophobic transmembrane regions, it is unlikely that LAMP2 is present as a soluble protein in the cytosol, therefore we assessed if LAMP2 could be part of a vesicle. Analysis of density gradient fractions revealed that LAMP2 appeared in lighter fractions in LLOMe exposed cells compared to untreated cells (Fig. 4c), indicating the presence of lysosomal membrane proteins in complexes smaller than the lysosome. To compare, the pattern for the transmembrane ER protein calnexin was not affected by LLOMe. By staining cytosolic extracts with the fluorescent membrane probe TMA-DPH, it was evident that LLOMe exposure caused an increased level of membranes positive for TMA-DPH as compared to the control (Fig. 4d). Furthermore, cytosolic LAMP2 was resistant to trypsin proteolysis (Fig. 4e). This suggests that LAMP2, which consists of a large intraluminal domain in addition to the transmembrane part and a small cytosolic domain, is protected from proteolysis inside a vesicle. A detergent would disintegrate these vesicles, making the LAMP2 accessible to trypsin. In support of this, addition of Triton X-100 reduced the level of LAMP2 upon trypsin digestion (Fig. 4e). Moreover, as shown in Fig. 4f, when performing immunoprecipitation with cytosolic LAMP2 as the bait, LAMP1 was co-precipitated, indicating that these two membrane proteins are present in the same structure. Taken together, these data suggest that LAMP2 found in the cytosol, as part of the cytoplasm, is present in vesicles smaller than the lysosome, in which the N-terminal peptide chain is directed to the lumen of the vesicle. We define the lysosomal membrane protein containing vesicle as lysosomal membrane protein complex (LMPC).

### ESCRT complex restores cell viability but does not contribute in the generation of LMPC

**Fig. 5:**
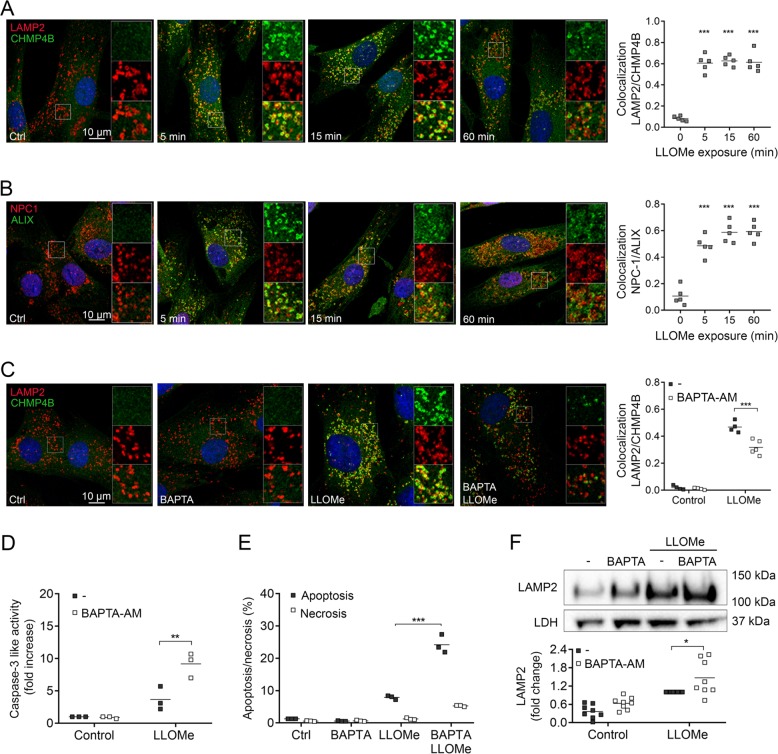
ESCRT complex recruited to LLOMe-damaged lysosomes promotes cell survival. Human fibroblasts were exposed to 1 mM LLOMe and when indicated, the cell permeable calcium chelator BAPTA-AM (1 µM) was added 10 min prior to LLOMe exposure. Immunocytochemical staining with corresponding colocalization analysis of **a** CHMP4B (green) and LAMP2 (red), **b** ALIX (green) and NPC-1 (red), and **c** CHMP4B (green) and LAMP2 (red) in fibroblasts exposed to LLOMe for 15 min. Nuclei are stained with DAPI (blue) and merged images show colocalization in yellow (*n* = 5). **d** Caspase-3 like activity in cells exposed to LLOMe for 8 h (*n* = 3). **e** Quantification of apoptotic and necrotic cells using Annexin V/PI staining in fibroblasts exposed to LLOMe for 6 h (*n* = 3). **f** Immunoblot of LAMP2 in digitonin-extracted cytosolic fractions from cells exposed to LLOMe for 2 h (*n* = 8) with corresponding quantitative analysis. *p < 0.05, **p < 0.01 and ***p < 0.001 compared to control (**a**–**b**) or LLOMe only (**c**–**f**).

Live-cell imaging of Lysotracker-stained cells showed that loss of fluorescence occurred within a minute after LLOMe exposure (Supplementary Video 1), demonstrating an instant damage to the lysosomal membrane. To test whether this damage stimulated ESCRT-induced repair, cells were co-stained with LAMP2 and the ESCRT-III-associated protein CHMP4B. Indeed, within 5 min after LLOMe-induced membrane disruption, CHMP4B was assembled on LAMP2-positive lysosomes (Fig. 5a). To further confirm the involvement of ESCRT, cells were also stained with ALIX and NPC-1. ALIX directs the ESCRT assembly to the injured membrane^27^, while NPC-1 is a lysosomal membrane protein that spans the membrane 13 times and does not appear in the cytosol upon LLOMe exposure (Supplementary Fig. 2e). Results show a similar pattern as for CHMP4B and LAMP2, establishing the recruitment of ESCRT to the damaged membrane (Fig. 5b). Addition of the membrane permeable calcium chelator BAPTA-AM confirmed that both CHMP4B and ALIX recruitment was calcium-dependent (Fig. 5c and Supplementary Fig. 2F). Moreover, when removing calcium, apoptosis was increased, as analysed by caspase-3 like activity (Fig. 5d) and Annexin V binding/propidium iodide staining (Fig. 5e), stressing the importance of a rapid membrane repair for cell survival. LAMP2 levels in the cytosol were not reduced, but slightly augmented, indicating that the ESCRT-induced membrane repair did not contribute to generation of LMPCs (Fig. 5f).

### Activation of lysophagy restores lysosomal function and promotes cell survival

**Fig. 6:**
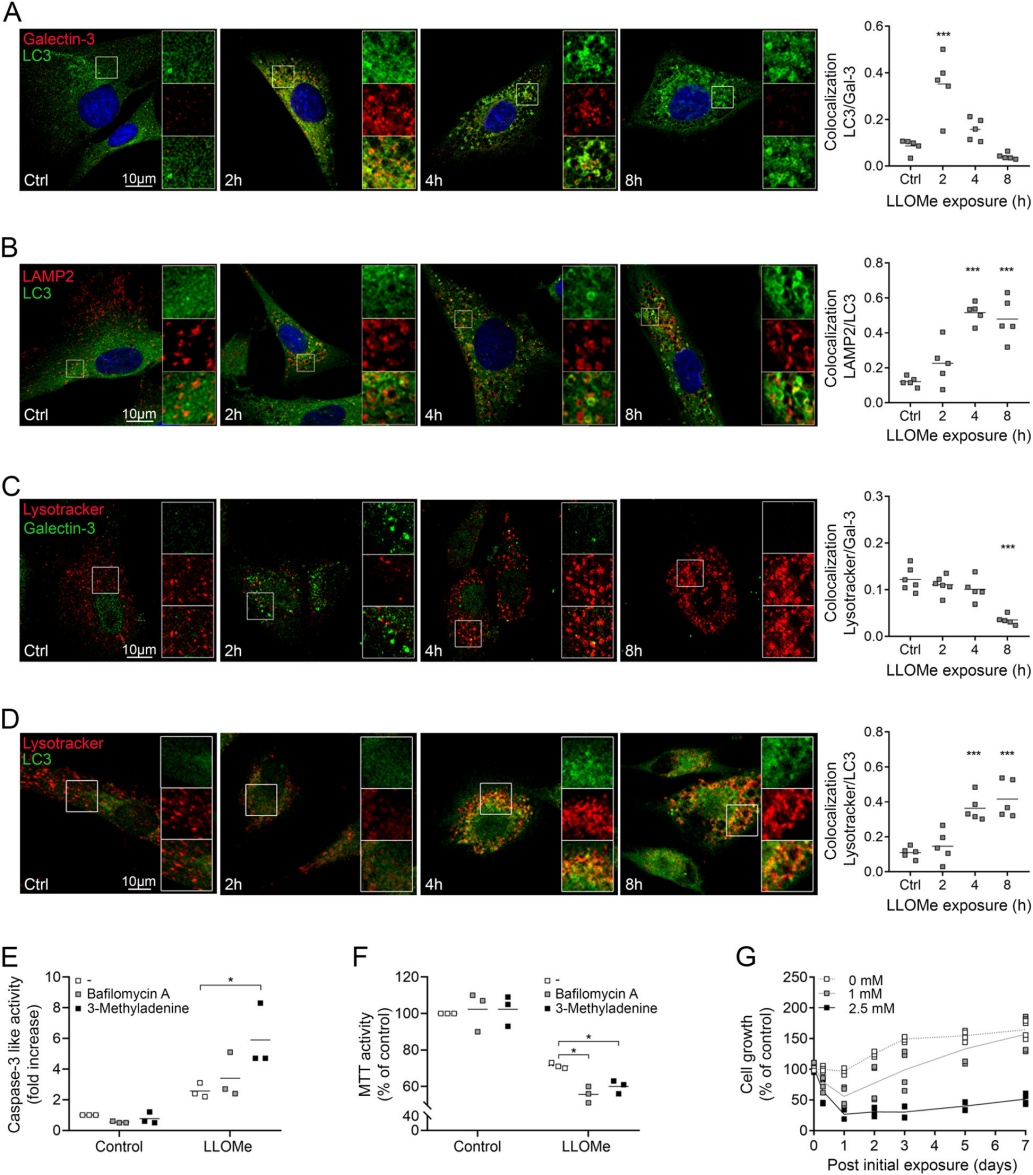
Lysophagic clearance restores lysosomal acidity and cell viability. Fibroblasts were exposed to 1 mM LLOMe and when indicated pretreated with the autophagy inhibitors 3-methyladenine (5 mM, 1 h) or bafilomycin A1 (25 nM, 30 min). Immunostaining with corresponding colocalization analysis of **a** LC3 (green) and galectin-3 (red), **b** LC3 (green) and LAMP2 (red), **c** Lysotracker (red) and galectin-3 (green), and **d** Lysotracker (red) and LC3 (green). Merged images show colocalization in yellow, nuclei are stained with DAPI (blue). **e** Caspase-3 like activity after 8 h of LLOMe exposure (*n* = 3). **f** MTT assay in fibroblasts exposed to LLOMe for 8 h (*n* = 3). **g** Crystal violet assay of cell viability for 0–7 days of cells exposed to LLOMe for 24 h (*n* = 4). *p < 0.05, **p < 0.01 and ***p < 0.001 compared to control (**a**–**d**) or LLOMe only (**e**–**f**).

Membrane repair by ESCRT is only efficient on small membrane perturbations, while larger disruptions require autophagic sequestration of the entire lysosome^15,16^. Staining of galectin-3 and the autophagy marker LC3 confirmed lysophagy in fibroblasts after LLOMe exposure (Fig. 6a). Image analysis show that LC3-positive vesicles colocalized with the galectin-marked lysosomes after 2 h of LLOMe exposure. Whereas LC3 increased in a time-dependent manner (quantified in Supplementary Fig. 3A), galectin-3 punctuate staining was reduced after 2 h of LLOMe exposure and almost abolished within 8 h (Fig. 6a, compare with quantification in Fig. 2b). The LC3 puncta also colocalized with LAMP2 (Fig. 6b), and together, these results verify the recruitment of LC3 to damaged lysosomes. Image analysis of colocalization between LC3 and LAMP2 present in lysosomes show that the main recruitment of LC3 occurred within 4 h of LLOMe exposure (Fig. 6b). We also observed concentration-dependent increase of the LC3II protein as shown by immunoblotting (Supplementary Fig. 3b), indicating that the activation of lysophagy was correlated to the extent of membrane damage. Image analysis revealed that the number of Lysotracker-positive lysosomes started to recover after a few hours (Fig. 6c, quantified in Supplementary Fig. 3C). Although galectin-3 recruitment was found to be localised to LAMP2-stained lysosomes (Fig. 2c), lysosomes positive for Lysotracker did not colocalize with galectin-3 puncta (Fig. 6c). Hence, Lysotracker only accumulated in acidic and intact lysosomes, in accordance with a previous report^19^. Sequestration of damaged lysosomes by lysophagy requires fusion with functional lysosomes to restore lysosomal pH. Indeed, in control cells there was no overlap between Lysotracker and LC3, but after LLOMe exposure, Lysotracker-positive vesicles were also positive for LC3 (Fig. 6d). At the same time, analysis of lysosomal pH confirmed that although the acidic pH was lost immediately after LMP induction, it was then restored within a few hours (Supplementary Fig. 3C). Inhibiting the recruitment of the autophagic machinery to damaged lysosomes by blocking the formation of autophagosomes with 3-methyladenine or by inhibiting lysosome-autophagosome fusion using the vacuolar V-ATPase inhibitor bafilomycin A1 resulted in a minor increase of apoptosis (Fig. 6e) and reduced cell viability (Fig. 6f), suggesting that lysophagic clearance of damaged lysosomes rescued cells from LMP-induced cell toxicity. The inhibition was confirmed by reduced co-staining of LAMP2 and LC3 (Supplementary Fig. 3E, F). Prolonged analysis of cell viability following LMP showed recovery and resumed proliferation at 1 mM LLOMe, but not after exposure to 2.5 mM (Fig. 6g), stressing the importance of lysosomal repair mechanisms for cell survival.

### Lysophagy is responsible for the generation of LMPC

**Fig. 7:**
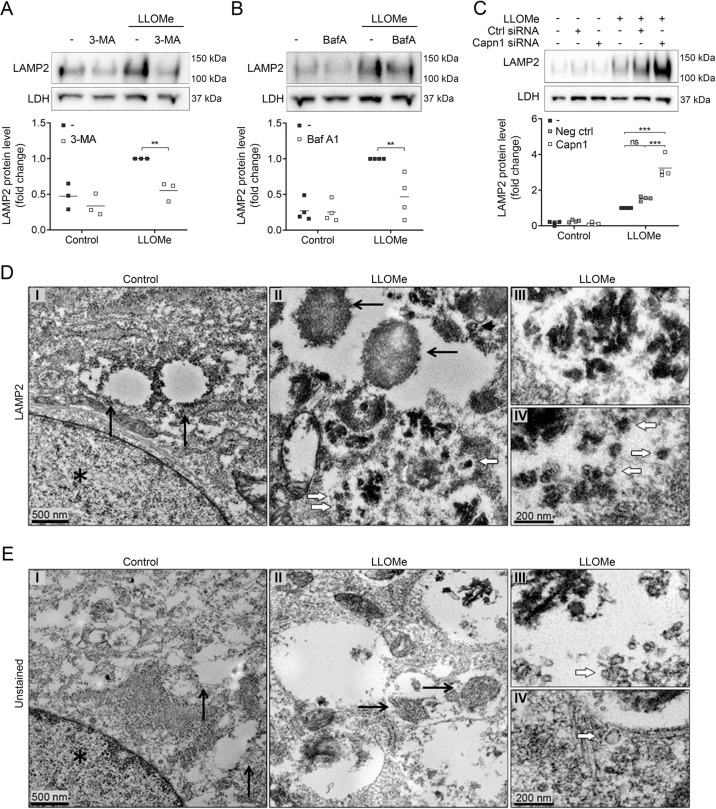
Lysophagy results in generation of lysosomal membrane protein complexes (LMPCs). Immunoblot with corresponding densitometric analysis of LAMP2 in digitonin-extracted cytosolic fractions from cells exposed to 1 mM LLOMe for 2 h after inhibition with **a** 3-methyladenine (3-MA, 5 mM, 1 h, *n* = 3), **b** bafilomycin A1 (Baf A, 25 nM, 30 min, *n* = 4) and **c** siRNA towards calpain-1 (10 nM, four different sequences). *p < 0.05, **p < 0.01 and ***p < 0.001. **d** Immuno-electron microscopy of LAMP2 with DAB development showing (I) control, (II) cell after 8 h of exposure to LLOMe and details of (III) lysophagosome and, (IV) cytosolic vesicles. **e** Non-DAB-stained specimens, (I) control, (II) cell 8 h after LLOMe addition and magnifications of (III) lysohagosome and, (IV) cytosolic vesicle. Black arrows indicate lysosomes, white arrows indicate small vesicles and *nucleus. Magnification x23,000 and ×36,000, scale bar 500 nm and 200 nm.

Next, we investigated the relationship between lysophagy and the appearance of LMPCs in the cytosol. By blocking with 3-methyladenine or bafilomycin A1, LAMP2 levels in the cytosol could be reduced (Fig. 7a, b). LLOMe has previously been shown to induce lysophagy via a galectin-3-dependent pathway^28^. In accordance, activation of lysophagy was impaired using anti-galectin-3, causing reduction of LAMP2 in the cytosol (Supplementary Fig. 3G). Co-staining of LC3 and LAMP2 confirmed that lysophagy was inhibited in the presence of anti-galectin-3, as the colocalization was significantly reduced (Supplementary Fig. 3F). Galectin-3-sensed lysophagy occurs via mobilisation of the autophagy regulator Beclin-1^17^. During apoptosis, calpains mediate cleavage of Beclin-1, resulting in a shift from autophagic to a more apoptotic phenotype, while knockdown of calpains prevents proteolytic cleavage of Beclin-1 and restores autophagy^29^. In line with this, we noticed that the cytosolic levels of LAMP2 increased in calpain-1 knockdowns (Fig. 7c), where calpain activity was reduced with ~50% (Supplementary Fig. 3H).

In order to investigate the LAMP2 location ultra-structurally, we performed transmission immuno-electron microscopy of DAB developed LAMP2 (Fig. 7d). For comparison negative control, not developed with DAB is included (Fig. 7e). In control fibroblasts DAB precipitate was detected along the borders of lysosomal structures (Fig. 7d(I), e(I), arrows). Eight hours after LLOMe exposure DAB precipitate was mainly found inside large vacuolar structures, indicating activation of lysophagy (Fig. 7d(II), e(II)). The material contained in the lysophages partly consisted of small vacuole-like structures, most clearly visualised in the DAB-negative images, due to covering of the structure by DAB precipitate in positive samples (compare 7d(III) and 7e(III)). In close vicinity to the lysophagic vacuoles, small vesicular structures with an approximate diameter of 50–100 nm and small precipitates on the border of the membrane were detected (Fig. 7d(IV), e(IV)). Thus, LAMP2-positive small vesicles, which might correspond to LMPCs, are detected both inside lysophagic vacuoles and as part of the cytoplasm. Micrographs at lower magnifications are shown in Supplementary Fig. 4 to present an overview of cell morphology.

In summary, we present evidence that moderate LMP induces lysosomal recovery mediated through an ESCRT-dependent repair mechanism and by lysophagic clearance. After lysosomal damage LAMP2 appears in the cytosol, present in a vesicle, LMPC. The generation of LMPCs is tightly concatenated with lysophagy since any manipulation of the efficiency of autophagy affects the LMPC levels. Although no protein synthesis of LAMP2 is detected, lysosomal function is restored, indicating that lysosomal material is recycled.

## Discussion

In this report, we study the effect of LLOMe-induced LMP and focus in particular on the presence of lysosomal membrane proteins in the cytosol. LLOMe-induced lysosomal membrane damage in human fibroblasts is followed by both ESCRT-dependent repair and lysophagy. Using several techniques, including immuno-electron microscopy, gradient fractionation and trypsin digestion, our results suggest that lysosomal membrane protein complexes (LMPCs) are generated and their appearance is correlated to lysophagy and cell survival.

Owing to the potential toxic effects of having lysosomal proteases in the cytosol^10^, cells harbour several mechanisms to protect against damaged lysosomes, and recently membrane repair by the ESCRT machinery or lysosomal clearance by lysophagy have been highlighted^30^. In accordance with previous findings^15,16^, ESCRT is rapidly recruited in a calcium-dependent manner upon loss of the lysosomal proton gradient. However, we find no significant calcium-dependent difference in the level of cytosolic LAMP2, indicating that the ESCRT mediated repair is not responsible for the generation of LMPCs. Even though LLOMe-induced lysosomal membrane insult causes loss of the proton gradient in a majority of cells, most cells do recover while the rest undergo apoptosis. The appearance of LMPCs coincides with activation of caspase-3. However, it is unlikely that the lysosomal membrane proteins, found in the cytosol, originate from apoptotic organelle fragments, as inhibition of apoptosis using a pan-caspase inhibitor does not affect the generation of LMPCs. Also, since LMPCs are detected hours after LMP-associated features, and appear concomitantly as the Lysotracker staining is regained, it is not likely that generation of LMPCs results from direct action of LLOMe on the lysosomal membrane.

Aberrant lysosomal function results in accumulation of undegraded material. To enhance cellular clearance, lysosomal biogenesis can be increased via activation of the master lysosomal regulator transcription factor EB (TFEB)^31,32^. Damage to the lysosomal membrane has been shown to induce nuclear translocation of TFEB and biogenesis of lysosomal proteins^17,33^. Newly synthesised lysosomal membrane proteins are post-translationally modified in the trans Golgi network before delivered either directly to lysosomes via endosomes^34^ or indirectly via the plasma membrane and re-internalisation via endocytosis^35^. In both these pathways, lysosomal membrane proteins are transported to their destination via vesicles. Therefore, it is possible that the LMPCs originate from vesicles containing newly synthesised membrane proteins en route to the lysosomal system. However, our data show that the total level of LAMP2 is retained and neither total protein synthesis nor LAMP2 synthesis is upregulated, but rather reduced. The half-life of LAMP1 has been shown to be 1.6 days in normal fibroblasts^36^ and Cuervo and Dice estimated the half-life of LAMP2 to be 44 h in cells subjected to starvation^37^, which is a condition that also induces lysosomal biogenesis via TFEB^38^. In accordance with our results, Maejima et al.^19^ analysed the total level of LAMP1 in cells treated with 1 mM LLOMe for 1 h followed by 25 µg/ml cycloheximide to inhibit protein synthesis. They found that the expression of LAMP1 was not affected by cycloheximide, not even after 10 h, and suggested that LAMP1 had a slow turnover in damaged lysosomes.

Interestingly, our results establish that the appearance of LMPCs is closely associated with activation of lysophagy. Damaged lysosomes are identified by galectin-3 binding, which is followed by autophagosome assembly and restoration of lysosomal pH. Galectins have been shown to elicit various response mechanisms, leading to phagophore formation, and are essential in cellular defence against bacterial infection by maintenance of membrane integrity^17,18^. Galectin-3 interacts with the tripartite motif protein TRIM16 and organises an ubiquitin-based autophagic response involving ATG16L1, ULK1 and Beclin-1^17^. Prevention of galectin-3 induced recruitment of the lysophagic machinery, as well as inhibition of autophagosome formation by 3-methyladenine and lysosomal-autophagosomal fusion by bafilomycin A, reduces the level of LAMP2 in the cytosol. Thus, inhibition of lysophagy prevents the generation of LMPCs. Accordingly, enhancing lysophagy by preventing proteolytic cleavage of Beclin-1 results in augmented LMPCs level. This indicates that lysophagic clearance of damaged lysosomes is responsible for the generation of LMPCs, via a galectin-3 and Beclin-1 induced autophagic pathway. It is important to note that even though 3-methyladenine and bafilomycin A are mainly used as autophagy inhibitors, other effects have been shown^39,40^.

Endosomal proteins are recycled back to biosynthetic and secretory organelles via retrograde trafficking^41^ and recently it was shown that the transmembrane protein Atg27 is recycled back from the yeast vacuole to the endosome and then to the Golgi^42^. Furthermore, Yu et al.^43^ have shown that although most lysosomes initially are consumed during starvation, they later recover in a process named autophagic lysosomal recovery (ALR). Upon ALR, tubular structures positive for lysosomal membrane proteins, but not containing any luminal content, are extended from autolysosomes. Subsequently, the tubules bud off LAMP1-positive vesicles that later mature into functional lysosomes, a process unaffected by the protein synthesis inhibitor cycloheximide, giving a possible mechanistic explanation to the LMPC studied here. In concordance, after 24 h the level of LMPCs in the cytosol is significantly reduced and fibroblasts resume proliferation.

**Fig. 8:**
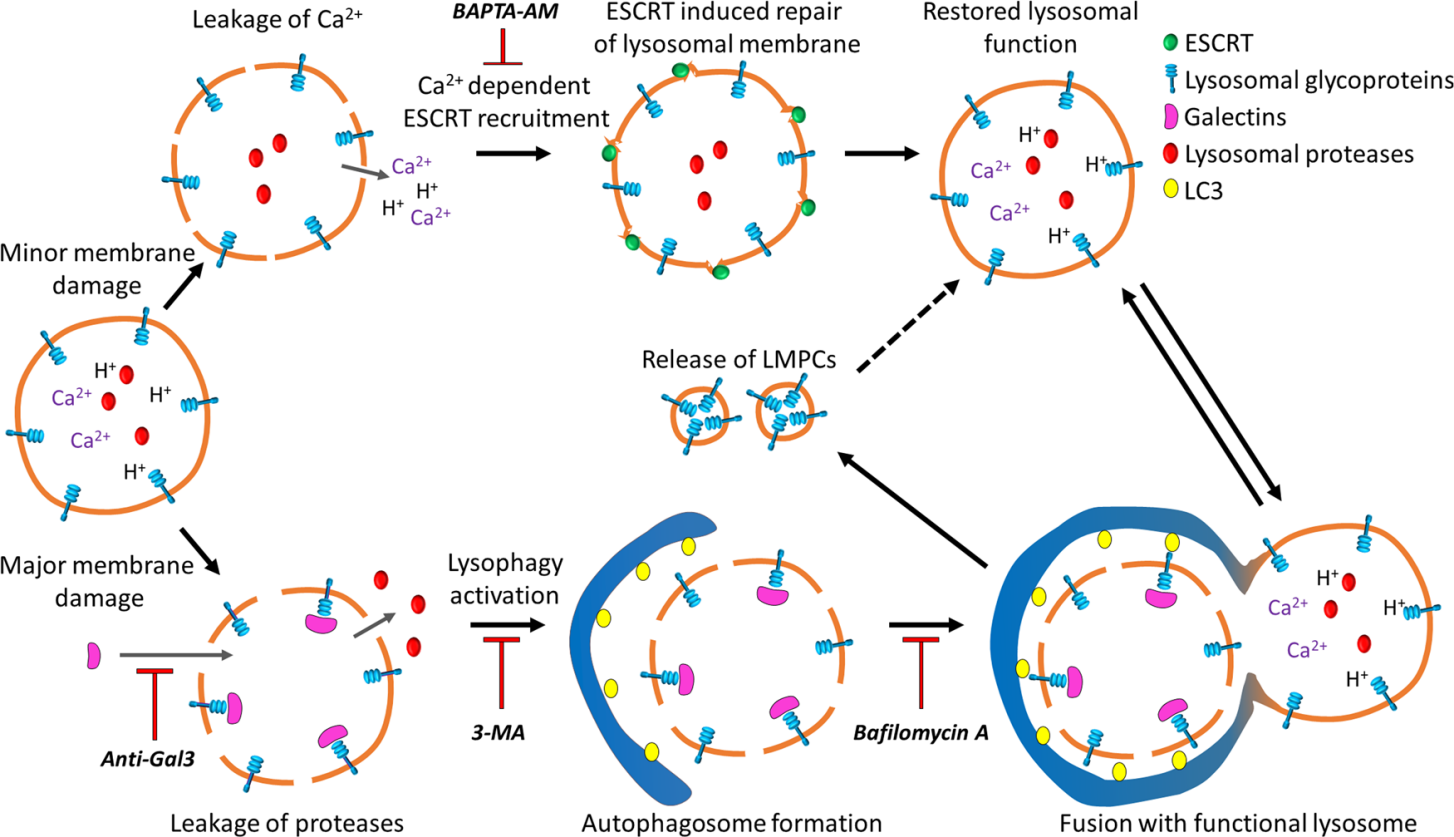
Graphical summary of lysosomal recovery mechanisms following LMP. Minor lysosomal membrane damage, causing release of protons and Ca^2+^ to the cytosol, activates membrane repair facilitated by ESCRT, which results in restored lysosomal function (upper part of image). More severe damage, causing a potentially detrimental release of lysosomal proteases, results in engulfment of the entire lysosome via lysophagy (bottom part of image). The lysophagic clearance generates release of lysosomal membrane protein complexes, LMPCs, probably to aid in restoration of lysosomal function.

We conclude that induction of LMP causes release of lysosomal content that impend cell viability, prompting the cell to regain a pool of functioning lysosomes. While ESCRT assembly at the lysosomal membrane repairs a fraction of the damaged lysosomes, lysophagy will be critical to sequester and degrade harmful organelles. Considering the slow turnover of lysosomal membrane proteins, a probable explanation for the formation of LMPCs is recycling of lysosomal membrane proteins, which enable efficient recovery of lysosomal activity and restoration of normal cellular function (Fig. 8).

## Materials and methods

### Cell culture and treatments

Human fibroblasts AG-1518 (passage 13-24, Coriell Institute, Camden, NJ, USA) were cultured in Eagle’s minimum essential medium supplemented with glutamax, 10% fetal bovine serum, 50 IU/ml penicillin-G and 50 μg/ml streptomycin (all from Gibco, Paisley, UK). Cells were authenticated and guaranteed mycoplasma free from the supplier and cultured for maximal two months. Primary keratinocytes were obtained from foreskin circumcisions of fair-skinned donors (0–3 years of age, parental written informed consent), established in cell culture and cultured for maximal 3 weeks as previously described^44^. The experiments were performed according to the ethical principles of the Helsinki declaration and approved by the Ethical Committee at Linköping University, Sweden. Cells were incubated in humidified air with 5% CO_2_ at 37 °C and were subcultured once a week. For experiments cells were trypsinized and seeded at a density that allowed them to reach 80% confluence at the time of LMP induction. Lysosomal membrane permeabilization was induced by exposing fibroblasts to the lysosomotropic agent LLOMe (0.1–10 mM) or MSDH (10–20 µM), and primary keratinocytes to UVA (340–400 nm, 60 J/cm^2^, Medisun 2000-L tube, Gröbel UV-Elektronik GmbH, Ettlingen, Germany) or UVB (280–370 nm, main output 305–320 nm, 500 mJ/cm^2^, Philips TL20W/12 tube, Philips, Eindhoven, The Netherlands). Apoptosis was induced using staurosporine (1 µM, 6 h). When indicated, cells were pretreated with the inhibitors Z-VAD-FMK (10 µM, 1 h), bafilomycin A (20 nM, 30 min), 3-methyladenine (5 mM, 1 h) anti-galectin-3 (5 µg/ml, 16 h, #556904, BD Pharmingen, San Diego, CA, USA) cycloheximide (20 µg/ml, 4 and 24 h) or BAPTA-AM (1 µM, 5 min). All chemical inhibitors were present during LMP induction. If not stated otherwise chemicals were obtained from Sigma-Aldrich, St. Louis, MO, USA. Depletion of calpain-1 was performed with four siRNA sequences (S102757314, S102757307, S102225552, S102225559; 10 nM of each) and 6 μl HiPerFect Transfection Reagent for 24 h (all from Qiagen, Germantown, MD, USA). AllStars negative control siRNA with a scrambled sequence without homology to mammalian genes (S103650318) was used as a negative control.

### Extraction of cytosol using digitonin

To analyse lysosomal release, cytosol was extracted using digitonin as previously described^45^. Extraction buffer (250 mM sucrose, 20 mM Hepes, 10 mM KCl, 1.5 mM MgCl_2_, 1 mM EGTA, 1 mM EDTA, 1 mM Pefabloc, pH 7.5) containing digitonin (25 µg/ml; Sigma-Aldrich) was added to cells and incubated on ice under agitation for 12 min, the extraction buffer was then removed and stored on ice. Crude organelle fractions were obtained by pelleting the remaining cells. For western blot analysis, cytosolic extracts were precipitated by addition of trichloroacetic acid (final concentration 5%). After 10 min on ice, the proteins were pelleted by centrifugation at 20,800 × *g* for 15 min. The pellets were then resuspended in lysis buffer (see below) containing 6 M urea and neutralised by the addition of 2 μl 1 M sodium hydroxide.

### Cell fractionation

Cells were resuspended in fractionation buffer (250 mM sucrose, 20 mM Hepes, 10 mM KCl, 1.5 mM MgCl_2_, 1 mM EGTA, 1 mM EDTA, 1X protease inhibitor cocktail) and then sonicated (4 × 15 s, 50% amplitude). For differential centrifugation, lysates were centrifuged at 720 × *g* for 5 min to pellet nuclei and cell debris. The remaining supernatant was centrifuged 20,000 × *g*, 4 °C for 5 min to pellet lysosomes and mitochondria, and the resulting supernatant was further centrifuged at 100,000 × *g*, 4 °C for 1 h, to collect the microsomal fraction. Remaining supernatant was considered the cytosolic fraction. For western blot analysis, the cytosolic fraction was concentrated using Amicon Ultra 3 K, 4000 × *g*, 4 °C, 40 min. For sucrose gradients, the postnuclear supernatant was loaded onto a discontinuous sucrose gradient (0.3–2 M) and centrifuged at 126,000 × *g*, 4 °C for 20 h. Fractions were collected using a Piston Gradient Fractionator (BioComp Instruments, Fredericton, Canada), precipitated by addition of trichloroacetic acid (final concentration 5%) and subjected to western blot analysis.

### Immunoblot analysis

Samples were lysed in 63 mM Tris-HCl, 10% glycerol and 2% sodium dodecyl sulfate (SDS). Protein content was determined using the Bio-Rad DC Protein Assay, and 5–10 µg aliquots of protein were loaded on a ClearPAGE SDS Gel (CBS Scientific, Del Mar, CA, USA) after addition of 50 mM dithiothreitol and 0.05% bromophenol blue. The proteins were transferred onto a nitrocellulose membrane using a iBlot 2 transfer device (Thermo Fisher Scientific, Waltham, MA, USA), followed by blocking for 90 min in Tris-buffered saline-Tween (TBS-Tween) (50 mM Tris, 0.15 M NaCl and 0.1% Tween-20, pH 7.5) containing 5% milk.

Primary antibodies, LAMP1 and LAMP2 (#9835-01 and 9840-01, Ms monoclonal, Southern Biotech, Birmingham, AL, USA), LIMP-II (ab176317, Rb monoclonal; Abcam, Cambridge, UK), LC3 (NB600-1384, Rb polyclonal, Novus Biologicals, Littleton, CO, USA), Calnexin (NBP1-85519, Rb polyclonal, Novus Biologicals), Cathepsin D (#01-12-030104, Rb polyclonal, Athens Research and Technology, Athens, GA, USA), NPC-1 (NB400-148, Rb polyclonal, Novus Biologicals), diluted in TBS-Tween containing 0.1% milk were added, and incubated at 4 °C overnight. The membrane was then washed and incubated for 1 h at room temperature with a horseradish peroxidase (HRP) conjugated anti-mouse or anti-rabbit antibody (P0447 and P0448, 1:3000; Dako, Glostrup, Denmark). For nascent protein analysis, HRP-conjugated Streptavidin (#21126, Thermo Fisher) was used to confirm newly synthesised proteins marked with biotin.

Equal loading was verified using an HRP-conjugated mouse anti-glyceraldehyde-3-phosphate dehydrogenase (GAPDH) antibody (NB300-328, Ms monoclonal, Novus Biologicals) for cell lysates and LDH (ab52488, Rb polyclonal, Abcam) for cytosolic extracts. Protein bands were visualised with Clarity ECL Substrate (Bio-Rad Laboratories, Hercules, CA, USA) and captured digitally using the Chemidoc XRS system (Bio-Rad Laboratories). Densitometric analysis was performed using the Image Lab Software (Bio-Rad Laboratories). The Chemidoc system alerts when pixels are saturated and only non-saturated blots were used for densitometric calculations. However, saturated bands are shown when samples from different cellular fractions were obtained in the same blot. Antibody concentration has been optimised to be within the linear range for the protein amount used.

### Immunoprecipitation

Immunoprecipitation was performed using the Pierce Classic Immunoprecipitation Kit according to the manufacturer’s instructions (Thermo Fischer Scientific). An equal volume of cytosolic extracts obtained by differential centrifugation were precleared for 45 min using Control Agarose Resin and incubated with 5 µg mouse anti human LAMP2 (#9840-01, Southern Biotech) or mouse isotype IgG antibody (X0931, DAKO) overnight at 4 °C. Immune complexes were then captured by incubation with Protein A/G Agarose Resin for 1 h and eluted in 2x SDS sample buffer and subjected to western blot.

### Nascent protein assay

Cells were pre-incubated in methionine- and serum-free media for 60 min and then incubated with 25 μM Click-iT AHA (l-azidohomoalanine, C10102, Molecular Probes, Eugene, OR, USA) with or without addition of LLOMe for 1 and 4 h, 37 °C. Cells were trypsinized, collected and lysed in 50 mM Tris-HCl, pH 8, containing 1% SDS and protease inhibitor (1x complete mini protease inhibitor cocktail) for 20 min on ice, followed by probe sonication (4 × 15 s). The samples were centrifuged 15,000 × *g* for 5 min, 4 °C and protein measured using the Bio-Rad DC Protein Assay. Sixty micrograms of protein was Click-IT ligated using Biotin conjugate and precipitated according to the manufacturer’s protocol (Molecular Probes). The samples were further processed for immunoprecipitation of biotin using Pierce Protein Streptavidin beads (Thermo Fisher Scientific) according to Pierce Classic IP Kit manual (Thermo Fisher Scientific). Precipitates were eluted in 2x SDS sample buffer and subjected to western blot.

### Trypsinization of membrane proteins

Cytosolic fractions obtained by digitonin extraction were mixed with 100–800 µg/ml trypsin. Samples were kept on ice and incubated on a rotator at slow speed for 15 min. Pefabloc (1 mM) was added and incubated for 5 min before protein precipitation using trichloroacetic acid (final concentration 5%) and samples were analysed by western blot as described for cytosolic extracts. As positive control, cytosolic fractions were incubated with Triton X-100 (1%, 20 min on ice) before subjected to trypsin digestion.

### Staining of membranes in digitonin-extracted cytosol using TMA-DPH

The presence of membrane fractions in the cytosol following LMP induction was analysed using 1-(4-trimethylammoniumphenyl)-6-phenyl-1,3,5-hexatriene p-toluenesulfonate (TMA-DPH). TMA-DPH is a hydrophobic fluorescent membrane probe, which partitions from aqueous solutions to incorporate into membranes, yielding an increased fluorescence signal. TMA-DPH (2 µM) was added to cytosolic extracts obtained using digitonin and incubated for 10 min at 37 °C. Fluorescence was then measured at *λ*_ex_ 355 nm/*λ*_em_ 460 nm using a Wallac 1420 Victor Plate Reader (PerkinElmer, Waltham, MA, USA) and correlated to LDH activity.

### Immunocytochemistry and image analysis

Cells cultured on glass coverslips were fixed in 4% paraformaldehyde for 20 min at 4 °C, incubated in 0.1% saponine and 5% fetal bovine serum (20 min, room temperature) followed by primary antibodies; LAMP2 (#9840-01, Ms monoclonal; Southern Biotech), LAMP2a (ab18528, Rb polyclonal), LC3 (NB600-1384, Rb polyclonal, Novus Biologicals), galectin-3 (#556904, Ms monoclonal; BD Pharmingen), NPC-1 (NB400-148, Rb polyclonal, Novus Biologicals), CHMP4B (13683-1-AP, Rb polyclonal; Proteintech, Rosemont, IL, USA), ALIX (MA1-83977, Ms monoclonal; Thermo Fisher Scientific) and IL-1β (ab9722, Rb polyclonal; Abcam), and the samples were incubated overnight in a humidified chamber at 4 °C. The cells were rinsed and incubated with secondary antibodies conjugated to Alexa Fluor dyes (Molecular Probes) for 1 h at room temperature. Coverslips were washed and mounted in ProLong Gold Antifade Reagent supplemented with 4´,6-diamidino-2-phenylindole (DAPI; Invitrogen, Paisley, UK). For staining of lysosomes, 100 nM Lysotracker Red DND-99 was added 30 min prior fixation (Thermo Fisher Scientific). The specimens were examined with a Zeiss laser scanning confocal microscope using 40x objective NA 1.3 (Carl Zeiss AG, Oberkochen, Germany). Optical section thickness was set to 0.9 µm (1 Airy unit). For all image analyses, 2D images from ≥4 randomly selected areas was obtained using the Zen software (Carl Zeiss AG). Colocalization in images was analysed using Pearson’s colocalization coefficient (Zen Imaging Software, Carl Zeiss AG) as the data was tested to be normal distributed and without outliers. This method was chosen over co-occurrence coefficients due to its measurement of relationship between signal intensities^46^. The threshold was set to exclude background staining for both channels and remained constant for all samples analysed. To quantify punctuate galectin-3 staining, specimens were blinded and at least 50 cells in randomly selected areas were quantified as either positive or negative for punctuate staining. The number of Lysotracker-positive puncta was analysed using the Huygens Imaging Software (SVI, Hilversum, Netherlands). For fluorescence intensity measurements, mean fluorescence was analysed in randomly selected areas using the Zeiss Zen Imaging Software (Carl Zeiss AG).

### Immuno-electron microscopy

Fibroblasts grown on coverslips were fixed in 4% formaldehyde and 0.05% glutaraldehyde in 0.1 M sodium phosphate buffer (pH 7.2; 20 min, 4 °C), incubated in 3% fetal bovine serum, 1% bovine serum albumin and 0.1% saponin (20 min, room temperature), followed by primary antibody (LAMP2, #9840-01, Ms monoclonal; Southern Biotech, overnight at 4 °C) and biotinylated secondary antibody (BA2020, Vector Laboratories, Burlingame, CA, USA; 1 h, room temperature). The biotinylated antibody was then visualised by incubating with Vectastain ABC HRP kit (Vector Laboratories; 1 h, room temperature) followed by DAB HRP substrate kit (Vector Laboratories) until staining was visible under light microscope. Cells were then post-fixed in 1% osmium tetroxide, dehydrated, en bloc stained with 2% uranyl acetate and embedded in epoxy embedding medium (Durcupan™ ACM Kit, Sigma-Aldrich). The blocks were sectioned using a Leica UC7 ultra microtome (Leica Microsystems GmbH, Vienna, Austria). Ultra-thin sections (60 nm) were cut with a diamond knife, collected onto formvar-coated copper slot grids, stained with lead citrate and examined in a Talos L120C transmission electron microscope (Thermo Scientific).

### Live-cell imaging

Cells were stained with Lysotracker Red DND-99 (100 nM, 30 min and Hoechst 33342 (2 µg/ml, 5 min) and visualised in a Zeiss laser scanning confocal microscope using 40x objective NA 1.3.

### Assessment of lysosomal membrane stability

Lysosomal membrane stability was analysed by digitonin extraction of cytosol followed by a measurement of cathepsin B and N-acetyl-β-d-glucosaminidase (NAG) activity. Sodium acetate buffer (0.1 M, pH 5.6) containing digitonin (25 and 200 µg/ml) was added to a 96-well plate and incubated for 10 min on ice with gentle agitation. The supernatant containing the cytosolic extract was then incubated at 37 °C for 40 min with 125 μl of 0.2 mol/l citrate buffer containing 0.8 mmol/l 4-methylumbelliferyl-2-acetamido-2-deoxy-β-d-glucopyranoside (NAG substrate; Sigma-Aldrich) or for 30 min with Z-Phe-Arg-AMC (30 µM; cysteine cathepsin substrate; Enzo Life Science, Farmingdale, NY, USA). The fluorescent product was determined using a Wallac 1420 Victor Plate reader (*λ*_ex_ 356 nm, *λ*_em_ 444 nm, and *λ*_ex_ 380 nm, *λ*_em_ 460 nm). The enzyme activity in each sample was correlated to the total enzyme activity obtained after cell membrane disruption using 200 µg/ml digitonin.

### Cell viability and caspase-3 like activity

Viability was analysed by the MTT (3-[4,5-Dimethylthiazol-2-yl]-2,5-diphenyltetrazolium bromide; Calbiochem, San Diego, CA, USA) reduction assay. Cells were incubated with 0.25 mg/ml MTT for 2 h at 37 °C. The MTT solution was removed and the formazan product dissolved in DMSO. Absorbance was analysed at 550 nm using a Wallac 1420 Victor Plate Reader. For analysis of long term viability, crystal violet assay was used. Following LLOMe exposure, cells were fixed in 4% paraformaldehyde at 4° and stained with crystal violet (0.04% in 1% ethanol) for 20 min at room temperature. The plate was washed, air dried and the crystal violet solubilized in 1% SDS. Absorbance was analysed at 550 nm using a Victor plate reader. Caspase-3 activity was analysed using the fluorescent substrate Ac-DEVD-AMC (Becton-Dickinson, Mountain View, CA, USA) according to the manufacturer’s instructions. Fluorescence was correlated to protein content, measured with Bio-Rad DC protein Assay.

### Flow cytometry of Annexin V/PI staining

The distribution of apoptotic, necrotic and viable cells was measured using the Dead Cell Apoptosis Kit with Annexin V Alexa 488 and propidium iodide (PI) (Thermo Fisher Scientific) according to the manufacturer’s instructions. Cells were trypsinized, washed with ice‐cold phosphate-buffered saline (PBS) and resuspended in 100 µl annexin-binding buffer, supplemented with 5 µl Alexa 488 Annexin V and 1 µg/ml PI for 15 min. Annexin-binding buffer (400 µl) was added and samples were immediately analysed in a flow cytometer equipped with a 488 nm argon laser (Gallios; Beckman Coulter, Indianapolis, IN, USA). Annexin V-positive cells were considered apoptotic and Annexin V/PI double-positive cells necrotic.

### LDH activity assay

Conditioned medium from cells exposed to LLOMe for specified time points were collected and transferred to a 96-well plate for LDH activity assay according to the manufacture’s description (Thermo Fisher Scientific). Briefly, LDH substrate mix was added to the medium (volume 1:1) and the plate was incubated at room temperature for 30 min. Stop solution was added to abort the reaction (volume 1:2) and absorbance was measured with a Wallac 1420 Victor Plate reader at 485 nm.

### Analysis of lysosomal pH using flow cytometry

Lysosomal pH was analysed using FITC-conjugated dextran as described previously^47^. Cells were allowed to endocytose FITC-conjugated dextran (0.1 mg/ml; 40 kDa; Sigma-Aldrich) for 72 h under standard culture conditions, followed by a 2-h chase period in fresh medium. Under this time cells were exposed to LLOMe. Cells were then trypsinized, resuspended in PBS and analysed in a flow cytometer equipped with a 488 nm argon laser. Fluorescence was measured in the FL1 channel using a SP 550 nm filter and a BP 525 ± 40 nm filter, and in the FL2 channel using a 595 SP and a BP 575 nm filter.

### Statistical analysis

Results from ≥3 independent experiments are shown as scatter plots presenting each data point with the mean value as a horizontal line. Statistical evaluation was performed with two-sided *t*-test between two groups and one-way ANOVA followed by Dunnett’s or Sidak’s multiple comparison post-test for >2 groups. Differences were considered significant at a *p* ≤ 0.05.

## Supplementary information

Supplementary Video 1

Supplementary Figure 1

Supplementary Figure 2

Supplementary Figure 3

Supplementary Figure 4

Supplementary Figure Legends
