# Supplementary figures and images for: Restoration of lysosomal function after damage is accompanied by recycling of lysosomal membrane proteins
Source: Cell Death Dis. 2020 May 14;11(5):370. doi: 10.1038/s41419-020-2527-8 (PMC7224388; doi:10.1038/s41419-020-2527-8)

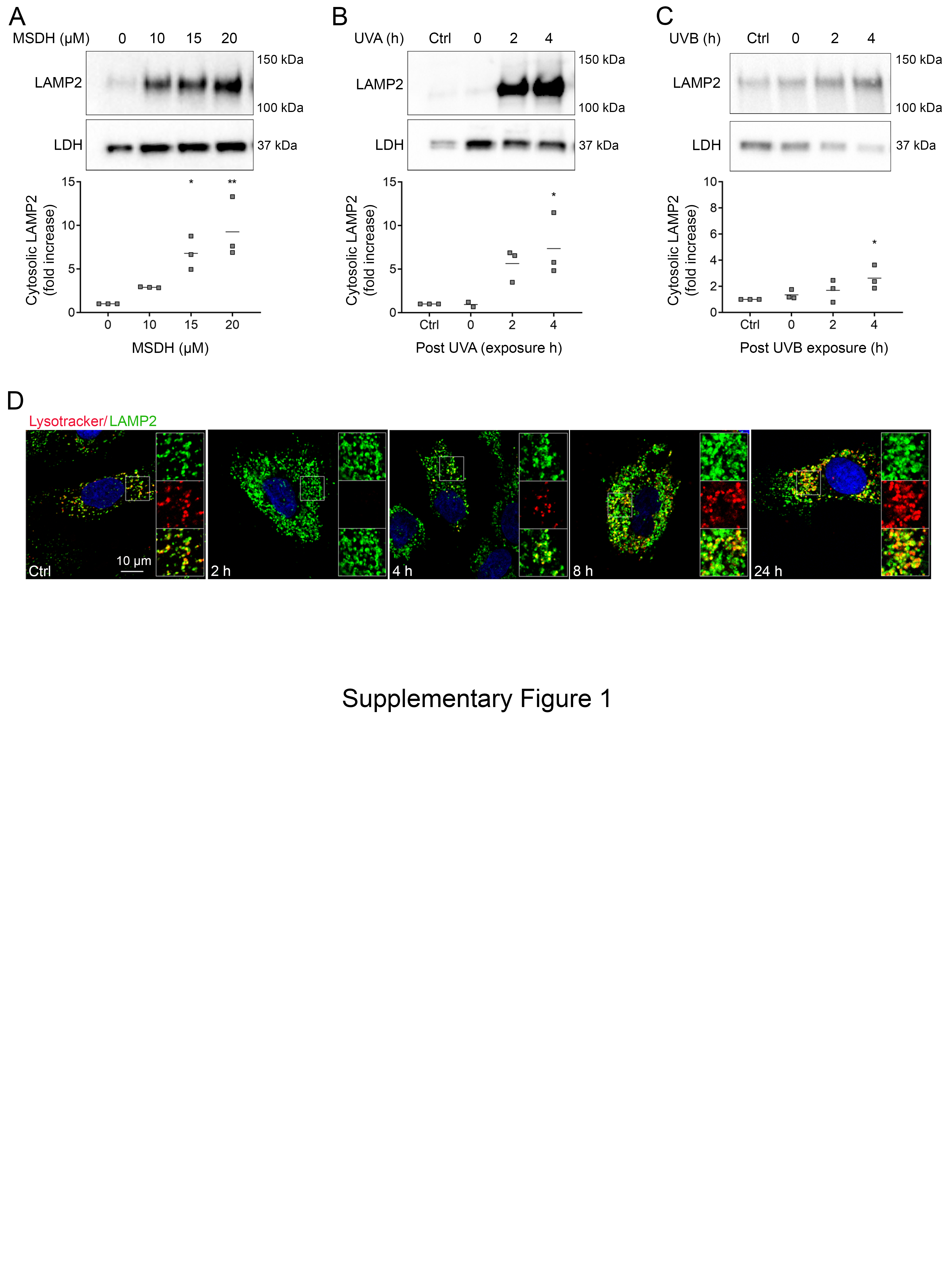

Supplement: Supplementary file 2 — Supplementary Figure 1 [file 41419_2020_2527_MOESM2_ESM.tif]

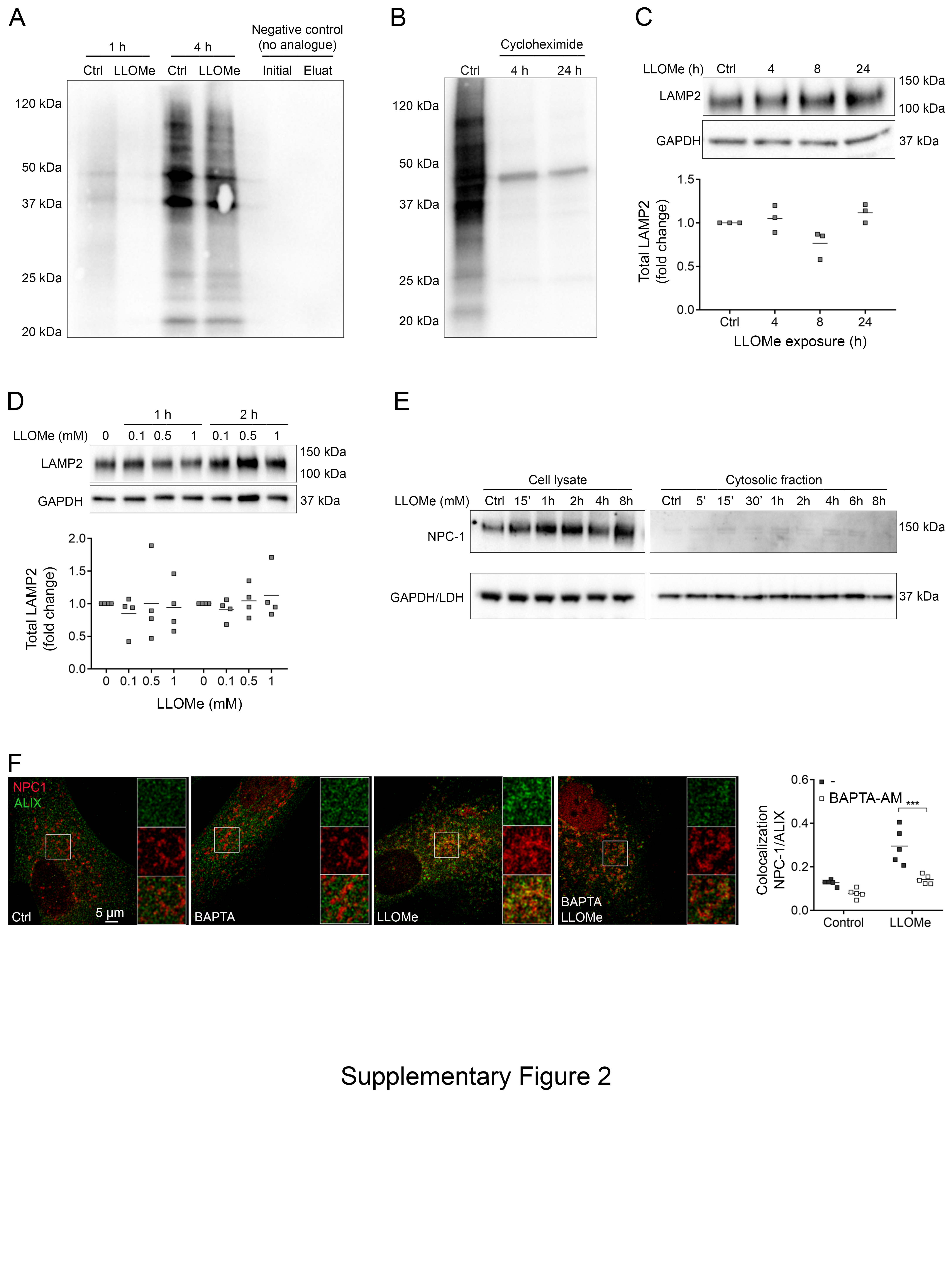

Supplement: Supplementary file 3 — Supplementary Figure 2 [file 41419_2020_2527_MOESM3_ESM.tif]

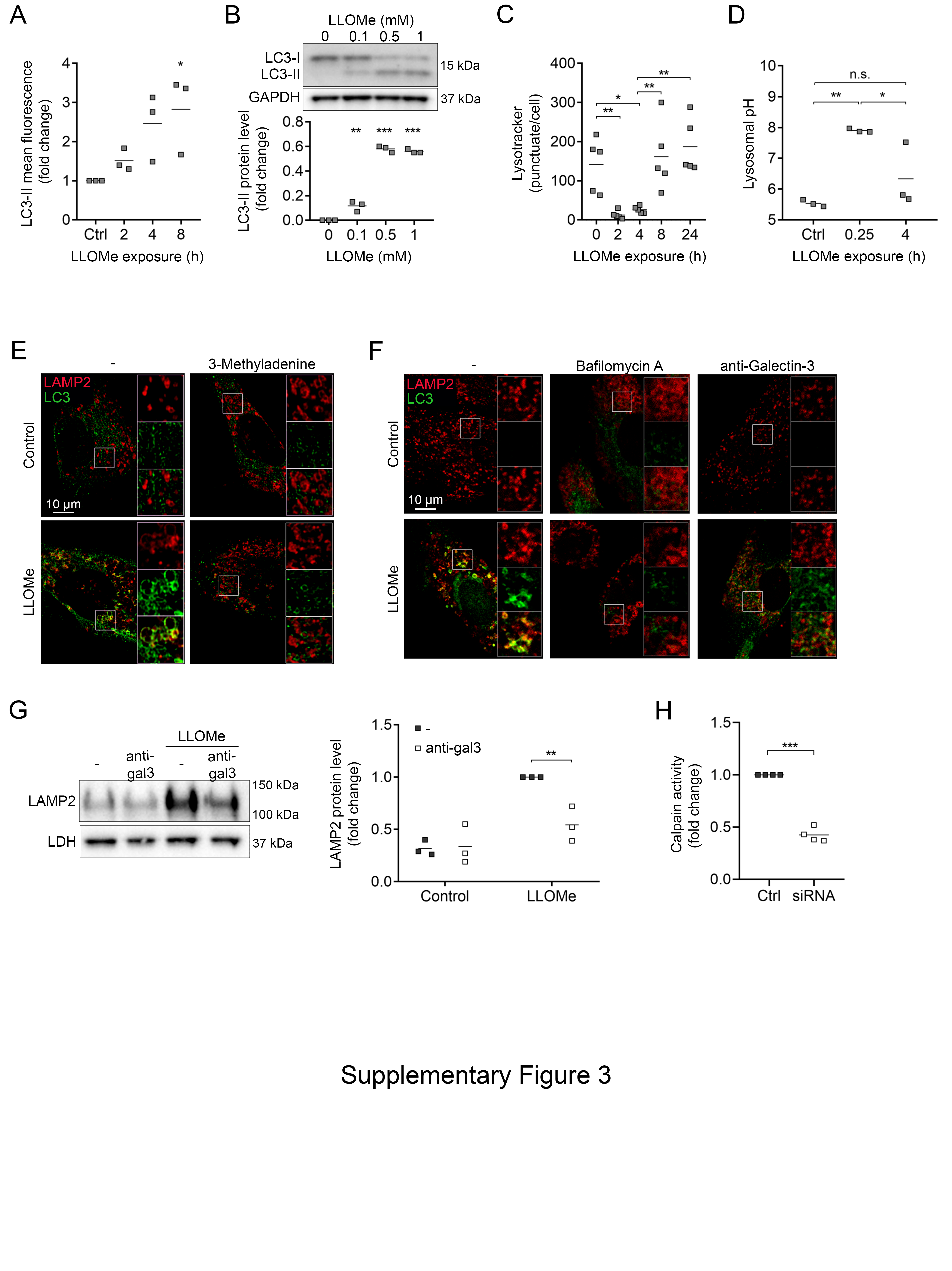

Supplement: Supplementary file 4 — Supplementary Figure 3 [file 41419_2020_2527_MOESM4_ESM.tif]

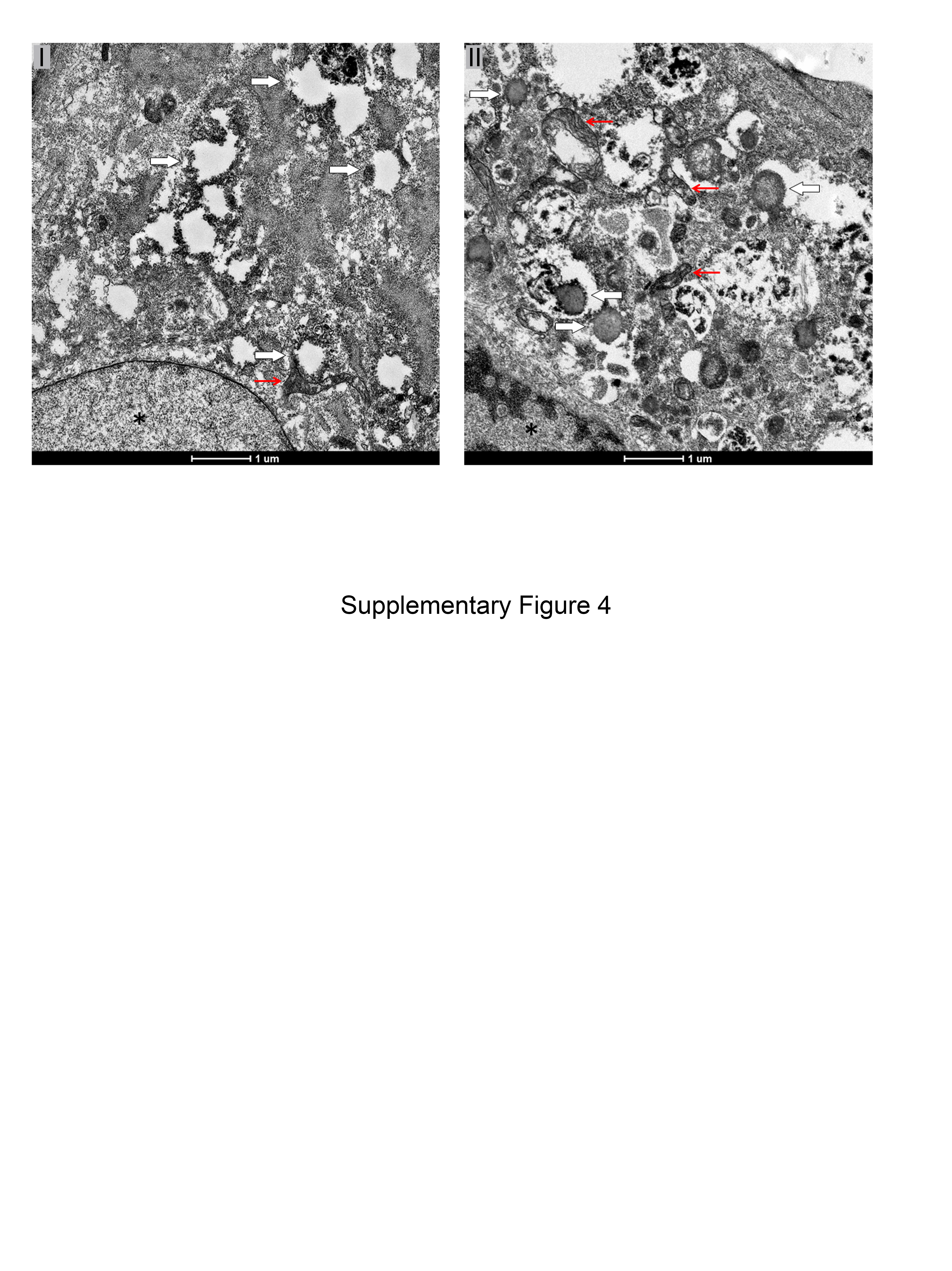

Supplement: Supplementary file 5 — Supplementary Figure 4 [file 41419_2020_2527_MOESM5_ESM.tif]
