# Supplementary material for: Restoration of lysosomal function after damage is accompanied by recycling of lysosomal membrane proteins
Source: Cell Death Dis. 2020 May 14;11(5):370. doi: 10.1038/s41419-020-2527-8 (PMC7224388; doi:10.1038/s41419-020-2527-8)
Supplement: Supplementary file 6 — Supplementary Figure Legends [file 41419_2020_2527_MOESM6_ESM.docx]

**Supplementary Figures and Video**

**Supplementary Figure 1. A)** Presence of cytosolic LAMP2 obtained by digitonin extraction in human fibroblasts exposed to 0-20 µM MSDH for 2 hours. Keratinocytes isolated from human skin irradiated with **B)** 60J/cm^2^ UVA and **C)** 500 mJ/cm^2^ UVB. Immunoblot of LAMP2 in digitonin extracted cytosolic fractions obtained immediately, 2 hours or 4 hours after irradiation. **D)** Immunostaining of LAMP2 (green), Lysotracker (red) and nuclei (blue) in fibroblasts exposed to 1 mM LLOMe. Merged images show colocalization in yellow. *p<0.05 and **p<0.01.

**Supplementary Figure 2. A)** Nascent proteins visualized using streptavidin in precipitated lysates from fibroblasts exposed to 1 mM LLOMe and co-incubated with a biotinylated methionine analogue for 1 or 4 hours. Negative controls incubated without methionine analogue before (initial) and after (eluate) precipitation are included. **B)** Method validation of nascent protein synthesis. Nascent proteins visualized using streptavidin in samples incubated with the protein synthesis inhibitor cycloheximide (20 µg/ml) for 4 and 24 hours. Immunoblotting of LAMP2 in whole cell lysates with corresponding densitometric analysis for **C)** time study after exposure to 1 mM LLOMe (n=3), and **D)** concentration dependence (n=4). **E)** Immunoblotting of NPC-1 in cell lysates and cytosolic extracts using digitonin after exposure to 1 mM LLOMe for 5 minutes to 8 hours. GAPDH is used as a loading control for lysates, LDH for cytosolic fractions. **F)** Immunocytochemical staining with corresponding colocalization analysis of ALIX (green) and NPC-1 (red) in human fibroblasts pretreated with the cell permeable calcium chelator BAPTA-AM (1 µM, 10 minutes) and then exposed to LLOMe (1 mM, 15 minutes). Merged images show colocalization in yellow. ***p<0.001.

**Supplementary Figure 3.** Human fibroblasts were exposed to 1 mM LLOMe at indicated time points. **A)** Quantification of LC3 mean fluorescence (n=3). **B)** Immunoblotting of LC3 in cell lysates obtained from fibroblasts exposed to 0-1 mM LLOMe for 2 hours with corresponding densitometric analysis (n=3). **C)** Quantification of Lysotracker puncta/cell (n=5). **D)** Analysis of lysosomal pH. **E and F)** Immunostaining of LAMP2 (red) and LC3 (green) in fibroblasts pretreated with 3-methyladenine (5 mM, 1 hour), bafilomycin A1 (25 ng/ml, 30 minutes) or anti-galectin 3 (5 µg/ml, 16 hours) and then exposed to LLOMe for 6 hours. **G)** Immunoblotting with corresponding densitometric analysis of LAMP2 in digitonin extracted cytosolic fractions from cells exposed to LLOMe for 2 hours after inhibition with anti-galectin 3 (anti-gal3, 5 µg/ml, 16 hours; n=3). **H)** Calpain activity measured using the Calpain activity Fluorometric Assay Kit (BioVIsion, Milpitas, CA, USA) in fibroblasts depleted of calpain-1. *p<0.05, **p<0.01, and ***p<0.001.

**Supplementary Figure 4.** Immuno electron microscopy micrographs of LAMP2 with DAB development showing (I) control and (II) cell after 8 hours of exposure to LLOMe. White arrows indicate lysosomes, red arrows mitochondria and * nucleus.

**Supplementary Video 1. Loss of lysosomal proton gradient is almost immediate after LLOMe addition.** Live cell imaging of fibroblasts stained with Lysotracker Red (purple, 100 nM, 30 minutes) and Hoechst 33342 (green, 2 µg/ml, 5 minutes) and exposed to 1 mM of LLOMe. Images were taken every 10^th^ second during 5 minutes.
